# Supplementary material for: Multilocus Genotyping and Intergenic Spacer Single Nucleotide Polymorphisms of Amylostereum areolatum (Russulales: Amylostereacea) Symbionts of Native and Non-Native Sirex Species
Source: J Fungi (Basel). 2021 Dec 11;7(12):1065. doi: 10.3390/jof7121065 (PMC8704056; doi:10.3390/jof7121065)
Supplement: Supplementary file 1 [file jof-07-01065-s001.zip › supplementary.pdf]

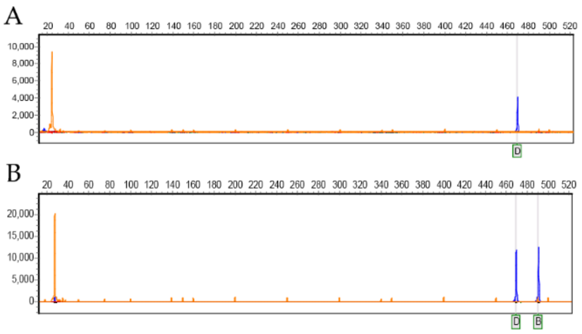

Figure S1. Fluorescence profiles for two IGS genotypes of *A. areolatum*. The peaks on the far right are IGS fragments. (**A**) Peaks for D alone, (**B**) peaks for B and D.

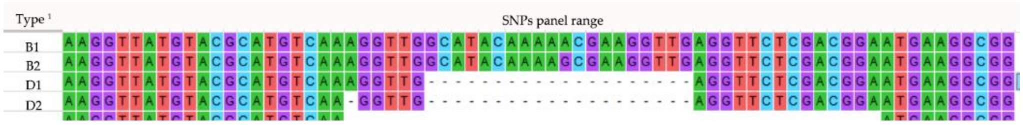

Figure S2. Multiple alignment of IGS haplotype sequences (type B and D) of the *Amylostereum areolatum* isolates from *Sirex nitobei* and *Sirex noctilio* specimens conducted in MEGA 7 software. The IGS sequences were obtained from the GenBank database, and alignment was performed to identify the SNP panel range (position 206 to 276) [48]. The IGS–SNP panel comprised the haplotypes B1, B2, D1, and D2 (from known IGS types B and D) denoted by [1] and the label boxes on the left. B1 and B2 were identified based on SNP at position 243, whereas D1 and D2 were identified based on SNP at position 227 in the haplotypes B and D, respectively.

Table S1. The intergenic spacer sequences included in the identification of SNP markers for detection of specific haplotypes in *Amylostereum areolatum* isolated from *Sirex* woodwasps

Woodwasp/Tree Host	Location/Country	Isolate Code	IGS Group
S. noctilio	New York, United States	AH-01	D1[1]
S. noctilio	New York, United States	GR94-11	B1D1[1]
S. sp. 'nitidus' (Harris)	Maine, United States	Sym-ME-09/10	B2E[1]
S. noctilio	Ontario, Canada	CMW36936	D2[1]
S. noctilio	Ontario, Canada	CMW36940	B1D2[1]
S. nigricornis	Ontario, Canada	CMW37009	B2E[1]
S. nigricornis	Ontario, Canada	CMW37037	E[2]
S. nigricornis	Louisiana, United States	ISOLATE20	E[2]
S. noctilio	New Zealand	DAOM 21785	AB[2]
S. noctilio	Tasmania	WaiteInst. 6195	AB[2]
S. noctilio	Brazil	Br17	AB[2]
S. noctilio	South Africa	M5W	AB[2]
S. juvencus	Denmark	DK782	AB[2]
S. juvencus	Lithuania	L204	AC[2]
S. juvencus	Sweden	S225	AC[2]
S. juvencus	Lithuania	L236	BC[2]
S. juvencus	Denmark	DK37	BC[2]

Deladenus siricidicola	Australia	A3	BC[2]
tree source (*Picea abies*)	Germany	CBS 334.66	C[2]
Unknown	France	CBS 305.82	D[2]
S. juvencus, S. noctilio, U. albicornis, U. gigas	Denmark, Hungary and Spain	RMK11-001	BC[2]
S.juvencus	Hungary	RMK11-011	BD[2]
S. nitobei	Japan	B1395	D2[1]
S. juvencus	Denmark	RMK11-006	BD[2]
S. juvencus, *S. noctilio*	Australia, Denmark, Hungary, Spain and US	GR94	BD[2]
tree source (*Picea abies*)	Germany	B1352	D[2]
S. juvencus	Germany	B1385	BD[2]
S. noctilio	US	AH1-17	D[2]
S. nitidus	US	SedDF	BE[2]
S. noctilio	Canada, South Africa and Chile	DAOM:239284	BD[2]
S. noctilio, *S. nitobei*	Dumeng, Jinbaotun, China	D18	B1D2[1]
S. noctilio	Dumeng, China	D3	B1D2[1]
S. nitobei	Linyi, China	L29	D2[1]
S. nitobei	Jinbaotun, Yushu, Linyi, China	M1	D2[1]
S. noctilio	Dumeng, Jinbaotun, Yushu, Hegang, China	D10	B1D2[1]

[1] IGS type was confirmed by cloning and sequencing of fragments.[2] IGS type was determined by fragment analysis of strains with either one or a combination of two haplotypes.
