# Supplementary figures and images for: Multilocus Genotyping and Intergenic Spacer Single Nucleotide Polymorphisms of Amylostereum areolatum (Russulales: Amylostereacea) Symbionts of Native and Non-Native Sirex Species
Source: J Fungi (Basel). 2021 Dec 11;7(12):1065. doi: 10.3390/jof7121065 (PMC8704056; doi:10.3390/jof7121065)

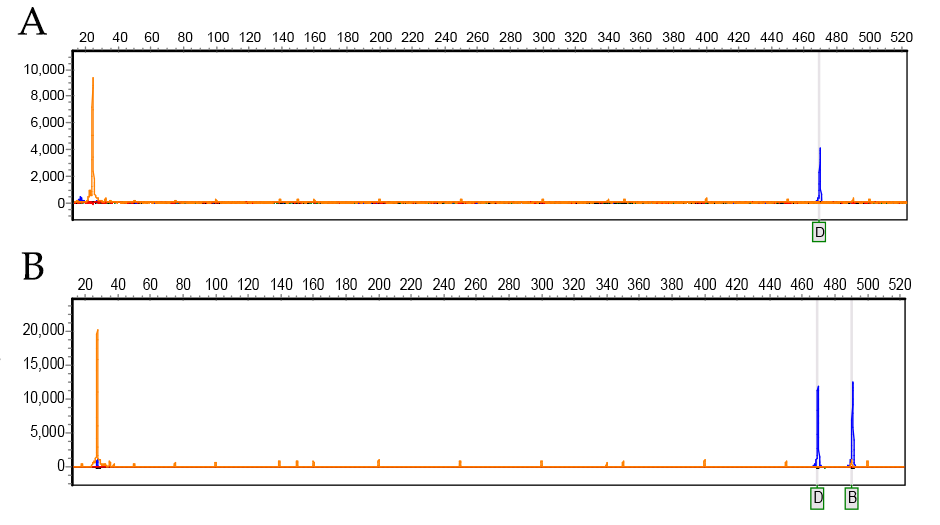

Supplement: Supplementary file 1 [file jof-07-01065-s001.zip › Figure S1.tif]

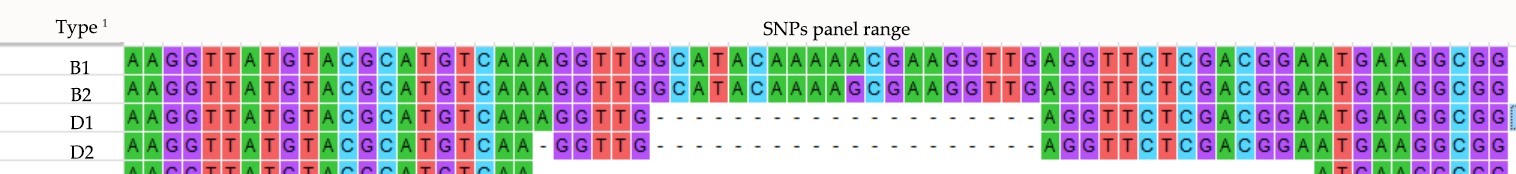

Supplement: Supplementary file 1 [file jof-07-01065-s001.zip › Figure S2.tif]
